# Supplementary material for: Coherent control of two Jaynes–Cummings cavities
Source: Sci Rep. 2024 Feb 15;14:3790. doi: 10.1038/s41598-024-53917-0 (PMC11303823; doi:10.1038/s41598-024-53917-0)
Supplement: Supplementary file 1 — Supplementary Information. [file 41598_2024_53917_MOESM1_ESM.pdf]

Coherent control of two Jaynes-Cummings cavities Supplementary Information

L. O. Castaños-Cervantes[1], Lorenzo M. Procopio[2], and Marco Enríquez[3,*]

[1]Tecnologico de Monterrey, School of Engineering and Sciences, Ciudad de Mexico 14380, Mexico

[2]Weizmann Institute of Science, Rehovot 7610001, Israel

[3]Tecnologico de Monterrey, School of Engineering and Sciences, Santa Fe 01389, Mexico
[*]menriquezf@tec.mx

A Some useful relations

In this section we explicitly write some of the equations used through out the core text. The expectation values in equation (25) are explicitly given as

$$\mathrm{Re}[\langle T_{ji}^{\dagger}\sigma_z T_{jj}\rangle] = \cos[(2t-t_m)g_j\sqrt{n_j+1}]\cos(t_m g_i\sqrt{n_i+1}), \tag{A-1}$$

and

$$\langle T_{ji}^{\dagger}\sigma_z T_{ji}\rangle = \cos[2(t-t_m)g_j\sqrt{n_j+1}]\cos^2(t_m g_i\sqrt{n_i+1}) - \cos[2(t-t_m)g_j\sqrt{n_j}]\sin^2(t_m g_i\sqrt{n_i+1}), \tag{A-2}$$

for $i \neq j$.

To compute the photon number and for non-identical cavities (28) the following relations are useful

$$\begin{aligned}
\langle T_{jj}^{\dagger}a_j^{\dagger}a_j T_{jj}\rangle &= \sin^2\left(g_j t\sqrt{n_j+1}\right) + n_j,\\
\langle T_{ii}^{\dagger}a_j^{\dagger}a_j T_{ii}\rangle &= n_j,\\
\mathrm{Re}[\langle T_{ij}^{\dagger}a_i^{\dagger}a_i T_{ii}\rangle] &= \cos\left(g_j t_m\sqrt{n_j+1}\right)\left[\sin\left(g_i t\sqrt{n_i+1}\right)\sin\left(g_i\left(t-t_m\right)\sqrt{n_i+1}\right) + n_i\cos\left(g_i t_m\sqrt{n_i+1}\right)\right],\\
\mathrm{Re}[\langle T_{ij}^{\dagger}a_j^{\dagger}a_j T_{ii}\rangle] &= n_j\cos\left(g_i t_m\sqrt{n_i+1}\right)\cos\left(g_j t_m\sqrt{n_j+1}\right),\\
\langle T_{ij}^{\dagger}a_i^{\dagger}a_i T_{ij}\rangle &= \sin^2\left[g_i\left(t-t_m\right)\sqrt{n_i+1}\right] + n_i,\\
\langle T_{ij}^{\dagger}a_j^{\dagger}a_j T_{ij}\rangle &= \sin^2\left(g_j t_m\sqrt{n_j+1}\right) + n_j,
\end{aligned} \tag{A-3}$$

for $i \neq j$. The coefficients ξ_l in equation (31) are explicitly given as

$$\begin{aligned}
&\xi_1 = \cos^3\theta e^{\phi_0'}e^{\phi_0}f_c(n_0+1,\Delta_0,g_0,t) + \cos\theta\sin^2\theta e^{\phi_0'}e^{\phi_1}f_c(n_0+1,\Delta_0,g_0,t-t_m)f_c(n_1+1,\Delta_1,g_1,t_m)\\
&\xi_2 = -\cos\theta\sin^2\theta e^{\phi_0'}e^{\phi_1}h_c(n_0+1,\Delta_0,g_0,t-t_m)f_c(n_1+1,\Delta_1,g_1,t_m) - h_c(n_0+1,\Delta_0,g_0,t)\cos^3\theta e^{\phi_0'}e^{\phi_0},\\
&\xi_3 = -\cos\theta\sin^2\theta e^{\phi_0'}e^{\phi_1}h_c(n_1+1,\Delta_1,g_1,t_m)f_c^*(n_0,\Delta_0,g_0,t-t_m),\\
&\xi_4 = \cos\theta\sin^2\theta e^{\phi_0'}e^{\phi_1}h_c(n_1+1,\Delta_1,g_1,t_m)h_c(n_0,\Delta_0,g_0,t-t_m),\\
&\xi_5 = e^{i\varphi}\cos^2\theta\sin\theta e^{\phi_1'}e^{\phi_0}f_c(n_0+1,\Delta_0,g_0,t_m)f_c(n_1+1,\Delta_1,g_1,t-t_m) + e^{i\varphi}\sin^3\theta e^{\phi_1'}e^{\phi_1}f_c(n_1+1,\Delta_1,g_1,t),\\
&\xi_6 = -e^{i\varphi}\cos^2\theta\sin\theta e^{\phi_1'}e^{\phi_0}f_c(n_0+1,\Delta_0,g_0,t_m)h_c(n_1+1,\Delta_1,g_1,t-t_m) - e^{i\varphi}\sin^3\theta e^{\phi_1'}e^{\phi_1}h_c(n_1+1,\Delta_1,g_1,t),\\
&\xi_7 = -e^{i\varphi}\cos^2\theta\sin\theta e^{\phi_1'}e^{\phi_0}h_c(n_0+1,\Delta_0,g_0,t_m)f_c^*(n_1,\Delta_1,g_1,t-t_m),\\
&\xi_8 = e^{i\varphi}\cos^2\theta\sin\theta e^{\phi_1'}e^{\phi_0}h_c(n_0+1,\Delta_0,g_0,t_m)h_c(n_1,\Delta_1,g_1,t-t_m),
\end{aligned} \tag{A-4}$$

where

$$f_c(n_j,\Delta_j,g_j,t) = \cos(t\Omega_j(n_j)) - \frac{1}{2}i\Delta_j\sin(\Omega_j(n_j)t)/\Omega_j(n_j), \quad h_c(n_j,\Delta_j,g_j,t) = -ig_j\sqrt{n_j}\sin(t\Omega_j(n_j))/\Omega_j(n_j),$$

and the operators $e^{\phi_j} = e^{-i\omega_j(n_j+1/2)t_m} e^{-i\omega_{j\oplus 1} a^\dagger_{j\oplus 1} a_{j\oplus 1} t_m}$ and $e^{\phi'_j} = e^{-i\omega_j(n_j+1/2)(t-t_m)} e^{-i\omega_{j\oplus 1} a^\dagger_{j\oplus 1} a_{j\oplus 1}(t-t_m)}$. Notice that, for the resonant case, i.e., $\Delta_0 = \Delta_1 = 0$, the phases e^{ϕ_j} will be cancelled when calculating the probabilities $|\xi_j|^2$.
